# Immunomodulatory effects of Blaps rynchopetera extract

**DOI:** 10.1590/acb370205

**Published:** 2022-05-02

**Authors:** Di Meng, Yinhe Yang, Longxing Li, Xiaoli Qian, Qi Wang, Jinglei Xu, Hairong Zhao, Heng Liu, Huai Xiao, Zhongtao Ding

**Affiliations:** 1 Master. Dali University – College of Pharmacy – Yunnan Provincial Key Laboratory of Entomological Biopharmaceutical R&D – Dali, China.; 2 PhD. Associate Professor. Dali University – College of Pharmacy – Yunnan Provincial Key Laboratory of Entomological Biopharmaceutical R&D – Dali, China.; 3 BS. Associate Professor. Dali University – Analysis and Testing Center – Dali, China.; 4 PhD. Professor. Dali University – College of Pharmacy – Yunnan Provincial Key Laboratory of Entomological Biopharmaceutical R&D – Dali, China.; 5 PhD. Professor. Dali University – College of Pharmacy – Dali, China, and Yunnan University – Functional Molecules Analysis and Biotransformation Key Laboratory of Universities in Yunnan Province – Kunming, China; Yunnan University, Functional Molecules Analysisand Biotransformation Key Laboratory, Kunming, China

**Keywords:** Insect, Macrophages, Immunocompromised Host, Mice

## Abstract

**Purpose:**

To explore the potential immunomodulatory effects of total extract and different polar parts from *Blaps rynchopetera* Fairmaire.

**Methods:**

Phagocytic activity was evaluated by neutral red assay, and the effect of the immune function was investigated by normal and immunocompromised mice models.

**Results:**

*In vitro*, total extract, as well as chloroform, ethyl acetate, n-butanol and water fractions could individually enhance the phagocytic ability of mouse peritoneal macrophages; in addition, chloroform and ethyl acetate fractions had an increasing tendency when combined stimulation with lipopolysaccharide (LPS). *In vivo*, ethyl acetate fraction (EAF) could enhance the immune organ index, increase the serum hemolysin level and peripheral blood immune cells of immunocompromised mice, while for normal mice, the effect was inconspicuous.

**Conclusions:**

*Blaps rynchopetera* extracts had noteworthy immunomodulatory effect, especially for individuals with immune disorders.

## Introduction

Immune regulation is one of the primary antitumor mechanisms of the traditional Chinese medicine. Studies have revealed that cancer and autoimmunity are closely related^1^^,^ 2, it can escape the immune system and even weaken the immune response of the host^3^^,^ 4. By activating the immunity of the body or regulating and balancing immune disorders, it can enhance physical fitness, inhibit tumor growth, improve patient survival time and quality of life, and even eliminate tumors^3^^,^ 5 ^,^ 6.

Insects not only have nutritional value but also have medicinal value^7^–^9^, and belong to a group of animal-based drugs widely used in traditional Chinese medicine. *Blaps rynchopetera* Fairmaire is one of the medicinal insects that belongs to the family Coleoptera Heteromera (Coleoptera) of beetles. It is also known as the *smelly fart bug* or *stink beetle* due to its smelly defensive secretion; it is used to treat fever, cough, gastritis, whitlow boils, tumors and other diseases^10^^,^ 11. There are reports documenting its medicinal properties such as its hypoglycemic^11^^,^ 12, antitumor^13^, antibacterial^14^, and antioxidant characteristics^15^. A key attribute of the traditional Chinese medicine is the diversity of its effective components and the synergistic effect of multiple targets, which is noticeably different from chemical drugs. If medicine can selectively inhibit the growth of tumor cells and also possess a good immunomodulatory activity, it may have a better therapeutic effect on the tumor.

This paper shows that the total extract, chloroform, ethyl acetate, N-butanol, and water fraction of *B. rynchopetera* could individually increase the phagocytosis index of mouse macrophages to varying degrees. Ethyl acetate fraction (EAF) boasts superior activity and can significantly relieve cyclophosphamide-induced immune deficiency in mice, increase the production levels of the hemolysin antibody, raise the level of immune cells in peripheral blood, and increase the spleen and thymus indexes. Results indicate that this fraction has a positive immune balance regulation effect.

## Methods

The experiments follow the rules of the school’s animal ethics committee, which approves all experiments for the purpose of controlling and supervising animal experiments (protocol number 2015-0820).

### Cells, reagents, experimental animals and materials

The following items were purchased from Beijing Solarbio Science and Technology Co., Ltd.: 3-(4,5-dimethylthiazol-2-yl)-2,5-diphenyltetrazolium bromide (MTT; Lot No. 303H0352), dimethyl sulfoxide (DMSO; Lot No. D5879), phosphate-buffered saline (PBS; Lot No. 11310221), neutral red (Lot No. 925B047), and 2-[4-(2-hydroxyethyl)-1-piperazinyl]-ethanesulfonic acid (HEPES; Lot No. 804D0411). Fetal bovine serum was purchased from Gibco, lipopolysaccharide (LPS) was purchased from Sigma, and Astragalus polysaccharide injection was purchased from Henan Fuxing Animal Pharmaceutical Co., Ltd. (Lot. No.20140615). Cyclophosphamide (Cytoxan, CTX) was purchased from Jiangsu Shengdi Pharmaceutical Co., Ltd. (Lot. No.16070425).

Healthy Kunming (KM) mice (18–22g), were purchased from Hunan Jingleike Jingda Experimental Animal Co., Ltd., license number: SCXX (Xiang) K2016-0002.

All the experimental samples were provided and prepared by Yunnan Provincial Key Laboratory of Entomological Biopharmaceutical R&D, Dali University.

### Mouse peritoneal macrophage phagocytosis assay

#### Preparation and purification of mouse peritoneal macrophages

After KM mice were sacrificed with cervical vertebra dislocation, macrophages were collected from the abdominal cavity, and the cell density was adjusted to 2 × 10^6^ cells·mL^–1^ with 1640 medium containing 10% fetal bovine serum. Next, 100 μL per well was inoculated on a 96-well plate and cultured at 5% CO_2_ and 37 °C. After 3 h, the unadherent cells were washed away with PBS to obtain purified peritoneal macrophages^16^^,^ 17.

#### Phagocytosis assay

Phagocytic activity was evaluated by neutral red assay^18^. The macrophage of 2 × 10^6^ cells·mL^–1^ was seeded in 96-well plates and set cell control group (cells and culture medium only) and LPS model group (LPS final concentration of 10 μg·mL^–1^). Respectively, sample groups (each with five concentration gradients of 500, 250, 125, 62.5, and 31.25 μg·mL^–1^) with LPS added (first stimulated with LPS for 4 h, then further stimulated with the addition of sample solution) were placed in three wells each. After culturing at 37 °C in 5% CO_2_ incubator for 24 h, washed the plate with PBS and added 200 μL neutral red solution to each hole, incubated for 3 h. The supernatant was discarded and the cells were washed with PBS. Then 200 μL cell lysate (containing 50% ethanol, 1% acetic acid, 49% water) was added and the solution was left at 4 °C overnight. Finally, the absorbance was measured at 520 nm. The phagocytic index was calculated as follows (Eq. 1):


$$\mathrm{Phagocytosis}\;\mathrm{index}\;\;=\;\frac{A\;\mathrm{sample}}{A\;\mathrm{blank}\;\mathrm{control}}$$ (1)

### Immune function assay for normal and immunocompromised KM mice

#### Preparation of sheep red blood cell (SRBC) suspension

Under aseptic conditions, blood was taken from the external jugular vein of healthy sheep, placed in an Erlenmeyer flask containing glass beads, and shaken for 30 min to remove fibrin. Next, PBS was added and the flask was stored in a refrigerator at 4 °C for later use. Before use, the sample was washed with normal saline and centrifuged for 5 min at 2000 r·min^–1^. After the supernatant was discarded, it was diluted with normal saline at a ratio of 3:5 (v/v) to obtain a red blood cell concentration of 2 million cells per microliter.

#### Preparation of guinea pig serum

Fresh blood was obtained from guinea pigs under aseptic conditions and the serum was separated and stored at –80 °C, then diluted with normal saline at 1:10 (v/v) before use.

### Grouping and administration

#### Normal mice model

The immune function assay for normal mice was performed in the same manner as previously described. The mice were randomly divided into six groups: control group, model group, Astragalus polysaccharides (APS) group, and three experimental groups (EAF-H, EAF-M and EAF-L). There were six mice in each group, half male and half female. Each subject in the blank group was given the same quantity of normal saline. The APS group was intraperitoneally injected with APS 160 mg·kg^–1^, while the experimental groups were given intragastric administrations of 400, 200, and 100 mg·kg^–1^. The medicine was administered once a day for 10 days. The model group was intraperitoneally injected 80 mg·kg^–1^ of CTX on the 7th day. Each sample group was immunized by an intraperitoneal injection of 0.2 mL 20% sheep red blood cell suspension on the 3rd, 5th, and 7th days of administration^19^.

#### Immunocompromised mice model

The immune function assay for immunocompromised mice was performed as previously described. The grouping and administration were essentially the same as for the normal mice model. Except for the control group, from the 7th day of administration, each group was injected intraperitoneally with 0.1 mL of 80 mg·kg^–1^ CTX and injected daily for three days to establish the model of immunosuppression in mice^20^.

#### Determination of peripheral blood immune cell levels

One hour after the last administration, blood was obtained from the eyeballs of the mice, and 20 μL of whole blood was placed in the prediluted solution. The peripheral blood cells were determined by an animal blood cell analyzer in prediluted mode.

#### Determination of serum hemolysin levels

The eyeball blood was centrifuged at 2000 r·min^–1^, taken the serum and inactivated at 56 °C for 30 min. Diluted and placed in an EP tube, added 0.5 mL of 5% sheep red blood cells and 1.0 mL of diluted guinea pig serum, normal saline as the blank control. All the test tubes were kept at 37 °C for 10 min with water bath and then put into ice bath to terminate the reaction. Centrifuged and took 1.0 mL of the supernatant, added 3.0 mL of Drabkin’s reagent; took another test tube and added 0.25 mL of sheep red blood cell suspension and 3.75 mL of Drabkin’s reagent^16^. After incubated for 10 min at room temperature, measure the absorbance of each sample at 540 nm and calculate the half hemolysis value (HC_50_) (Eq. 2)^21^:


$${\mathrm{HC}}_{50}\;\;=\;\frac{\mathrm{Absorbance}\;\mathrm{value}\;\mathrm{of}\;\mathrm{sample}}{\mathrm{Half}\;\mathrm{absorbance}\;\mathrm{value}\;\mathrm{of}\;\mathrm{red}\;\mathrm{blood}\;\mathrm{cells}\;\mathrm{during}\;\mathrm{half}\;\mathrm{hemolysis}}\;\times \;\mathrm{Dilution}\;\mathrm{multiple}$$ (2)

#### Determination of organ index

The mice were sacrificed after blood collection, then the spleen and thymus were removed and accurately weighed to calculate the organ index of the immune organs (Eq. 3).


$$\mathrm{Organ}\;\mathrm{index}\;\;=\;\frac{\mathrm{Organ}\;\mathrm{mass}\;(\mathrm{mg})}{\mathrm{Mice}\;\mathrm{body}\;\mathrm{weight}\;(g)}$$ (3)

### Statistical analysis

A one-way analysis of variance was performed with SPSS 19.0 software. After the homogeneity of the variance test, the experimental data with uniform variance were statistically analyzed by the pairwise comparison Fisher’s least significant difference method. Besides, the data with uneven variance was analyzed by the rank sum test. Values of p < 0.05 were considered statistically significant.

## Results

### Effect of B. rynchopetera on phagocytic ability of peritoneal macrophages

Mouse peritoneal macrophages were used as the experimental object and LPS acted as a positive control. The phagocytic ability of macrophages was investigated by single sample stimulation and sample combined with LPS stimulation^22^. The immunological activity of the total extract, chloroform, ethyl acetate, n-butanol, and water fractions of *B. rynchopetera* were tested.

**Figure 1:**
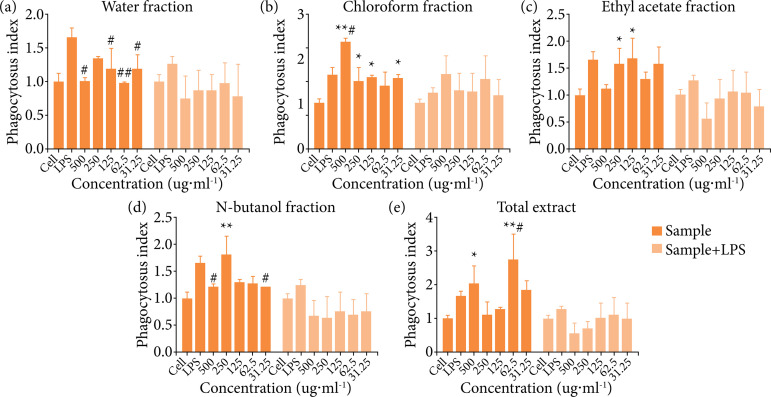
Effect of different excerpts from *B. rynchopetera* on phagocytosis of the macrophages.

The results are displayed in Fig. 1 (the left parts). The phagocytosis of mouse peritoneal macrophages was significantly enhanced under the stimulation of LPS, reflected by a phagocytosis index was greater than 1.0 (p < 0.05). Except the water fraction, the total extract and three different polar fractions, i.e., the chloroform, ethyl acetate, and n-butanol fractions, all could enhance the phagocytic ability of macrophages. There were significant differences (p < 0.05) for some fractions at certain concentration, particularly for the chloroform fraction and EAF, while there were no dose-dependent.

When stimulated with sample combined with LPS (the right parts of Fig. 1), only the chloroform fraction showed a trend of enhancing macrophage phagocytosis, the total extract, and other fractions had no synergistic enhancement. On the contrary, all dose groups of n-butanol and water fractions acted a certain inhibitory activity when combined with LPS stimulation.

Compared with cell group, *p *<* 0.05, **p < 0.01; Compared with LPS group, ^#^p < 0.05, ^##^p < 0.01.

### Immune function effect of EAF on normal and immunocompromised KM mice

#### Effect of EAF on immune organs index

In the immune normal model, CTX was used as a model control and APS as a positive control. In the immunocompromised group, except for the control group, all mice were injected intraperitoneally with CTX, and APS was also used as the positive control.

The immune organs index can be used to determine the immune function of animals, while the spleen and thymus indexes can reflect the level of immune response to antigens.

**Figure 2:**
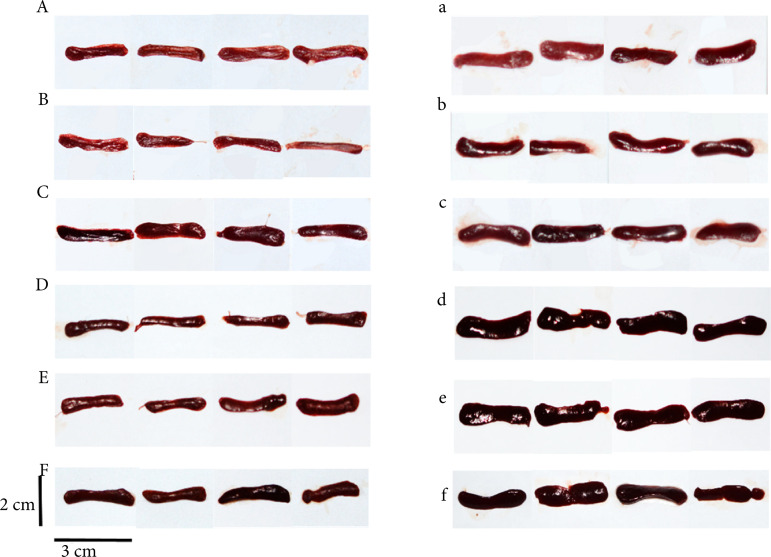
Representative spleens of experiment; A–F represented the control, CTX, APS, and EAF high, middle, low doses groups of immune normal mice and a–f represented immunocompromised mice experiment respectively.

The results in Table 1 and Fig. 2 showed the effect of immune organs index in normal mice. Compared with control group, the liver, spleen and thymus indexes of model group (CTX) showed a slightly decreasing trend, but there was no statistical significance. Compared with the model group, the spleen index of all the experimental groups increased to some extent, but only the APS group and EAF low-dose group displayed statistical differences (p < 0.01), thymus index of all the experimental groups showed an increasing trend, but only the APS group had statistical differences (p < 0.01). As shown in Fig. 2, the changes of spleen in each group were consistent with those in Table 1, but the color was not uniform, which might be caused by different exposures during photography.

**Table 1:** Effect of EAF on immune organ index of normal mice ( ± s, n = 6).

Groups	Dose (mg·kg^–1^)	Spleen index (mg·g^–1^)	Thymus index (mg·g^–1^)
Control	-	2.07 ± 0.46	1.23 ± 0.68
CTX	-	1.72 ± 0.53	0.72 ± 0.50
APS	160	5.68 ± 0.63** ∆∆	1.74 ± 0.54∆∆
EAF-H	400	2.27 ± 0.60▲▲	1.04 ± 0.99
EAF-M	200	2.64 ± 1.23▲▲	1.15 ± 0.79
EAF-L	100	3.55 ± 0.98** ∆∆ ▲▲	0.93 ± 0.66▲

* p < 0.05

** p < 0.01

compared with the CTX group

∆ p < 0.05

∆∆ p < 0.01

compared with APS group

▲ p < 0.05

▲▲ p < 0.01.

**Figure 3:**
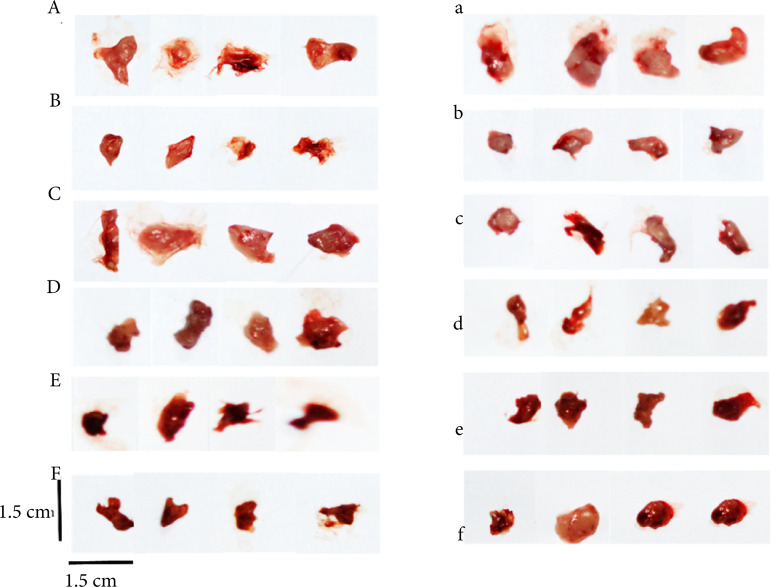
Representative thymuses of experiment; A–F represented the control, CTX, APS, and EAF high, middle, low doses groups of immune normal mice and a–f represented immunocompromised mice experiment respectively.

Table 2 and Fig. 3 showed the effect of the immune organs index in immunocompromised mice. Compared with the control group, the spleen index and thymus index of model group (CTX) were significantly reduced (p < 0.01). Also, compared with the model group, the spleen index of all the experimental groups were significantly increased (p < 0.01), and even exceeded the control group (p < 0.01). However, there was no statistical difference in the thymus index.

**Table 2:** Effect of EAF on immune organ index in immunocompromised mice ( ± s, n = 6).

Groups	Dose (mg·kg^–1^)	Spleen index (mg·g^–1^)	Thymus index (mg·g^–1^)
Control	-	3.82 ± 0.77	2.93 ± 0.92
CTX	-	2.33 ± 0.57**	1.11 ± 0.41**
APS	160	6.07 ± 1.13** ∆∆	1.28 ± 0.44**
EAF-H	400	7.73 ± 2.62** ∆∆	1.17 ± 0.36**
EAF-M	200	6.17 ± 3.18** ∆∆	1.47 ± 0.40**
EAF-L	100	7.63 ± 3.72** ∆∆	1.53 ± 0.42**

Compared with control group, * p < 0.05

** p < 0.01

compared with the model group

∆ p <0.05

∆∆ p *<* 0.01

compared with APS group.

### Effect on peripheral blood immune cell levels

Variations in peripheral blood cells can reflect the changes of immune function^23^. The effect on peripheral blood immune cell levels for normal mice were showed in Table 3. Compared with the control group, the levels of white blood cells, lymphocytes, monocytes and granulocytes in the peripheral blood of mice in the model group (CTX) had a decreased tendency, and the decrease of lymphocytes was statistically significant (p < 0.01). The presence of APS can significantly increase the levels of these four immune cells in normal mice (p < 0.01). Additionally, the levels of leukocytes, lymphocytes and monocytes in all dose groups of EAF showed an increasing trend. However, only the levels of lymphocytes and monocytes in the high dose group had a significant difference (p < 0.05), and there was no effect on granulocyte level.

**Table 3:** Effect of EAF on immune cells in peripheral blood of normal mice ( ± s, n = 6, 1 × 10^9^/L).

Groups	Dose (mg·kg^–1^)	Leukocyte	Lymphocytes	Monocyte	Granulocyte
Control	-	1.26 ± 0.68	0.59 ± 0.18	0.04 ± 0.03	0.63 ± 0.59
CTX	-	0.77 ± 0.37	0.33 ± 0.13**	0.03 ± 0.01	0.41 ± 0.25
APS	160	3.23 ± 1.05** ∆∆	1.59 ± 0.82** ∆∆	0.13 ± 0.03** ∆∆	1.52 ± 0.44** ∆∆
EAF-H	400	1.75 ± 0.51∆∆▲▲	1.06 ± 0.37** ∆∆	0.09 ± 0.04* ∆∆	0.60 ± 0.20▲▲
EAF-M	200	1.27 ± 0.13∆∆ ▲▲	0.71 ± 0.06∆∆ ▲	0.08 ± 0.06∆ ▲	0.48 ± 0.04▲▲
EAF-L	100	1.40 ± 0.43∆∆ ▲▲	0.63 ± 0.13∆∆ ▲▲	0.08 ± 0.04∆ ▲	0.68 ± 0.32▲▲

Compared with control group,

* p < 0.05

** p < 0.01

compared with CTX group

∆ p < 0.05

∆∆ p < 0.01

compared with APS group

▲ p < 0.05

▲▲ p < 0.01.

The effect on immunocompromised mice was very different, as Table 4 illustrates. Compared with the control group, the levels of leukocytes and lymphocytes in CTX group were significantly decreased (p < 0.01). Moreover, levels of the four kinds of immune cells in all the experimental groups were significantly increased (p < 0.01), compared with the model group. Additionally, the levels of white blood cells, monocytes, and granulocytes were higher, exceeding the control group (p < 0.01).

**Table 4:** Effect of EAF on immune cells in peripheral blood of immunocompromised mice ( ± s, n = 6.1 × 10^9^·L^-1^).

Groups	Dose(mg·kg^–1^)	Leukocyte	Lymphocytes	Monocyte	Granulocyte
Control	-	1.46 ± 0.28	1.14 ± 0.27	0.04 ± 0.01	0.27 ± 0.05
CTX	-	0.72 ± 0.37**	0.31 ± 0.14**	0.03 ± 0.01	0.37 ± 0.24
APS	160	3.09 ± 0.75** ∆∆	1.33 ± 0.42∆∆	0.13 ± 0.03** ∆∆	1.55 ± 0.47** ∆∆
EAF-H	400	2.26 ± 0.19** ∆∆ ▲	0.83 ± 0.16∆∆ ▲▲	0.26 ± 0.07** ∆∆ ▲▲	1.17 ± 0.30** ∆∆
EAF-M	200	2.26 ± 0.84* ∆∆ ▲	0.70 ± 0.27* ∆∆ ▲▲	0.12 ± 0.04** ∆∆	1.45 ± 0.65** ∆∆
EAF-L	100	2.30 ± 0.48** ∆∆ ▲	0.85 ± 0.21∆∆ ▲▲	0.15 ± 0.03** ∆∆	1.04 ± 0.31** ∆∆ ▲

Compared with control group,

* p < 0.05,

** p < 0.01;

compared with the model group,

∆ p < 0.05

∆∆ p < 0.01;

compared with APS group,

▲ p < 0.05

▲▲ p < 0.01.

#### Effect of EAF on serum hemolysin levels in mice

Serum hemolysin level is one of the commonly-used indicators that evaluates humoral immune response, which reflects the level of antibodies^24^.

Table 5 showed the effect of serum hemolysin levels in normal mice. Compared with the control group, the HC_50_ of the CTX group was significantly reduced (p < 0.01), while the positive drug APS group was significantly increased (p < 0.01). The EAF exhibited an increase, with the high and medium dose groups presenting a slight rising trend, but only the low-dose group displayed statistical significance (p < 0.05).

**Table 5:** Effect of EAF on the level of serum hemolysin production in normal mice ( ± s, n = 6).

Groups	Dose (mg·kg^–1^)	Half hemolysis value (HC_50_)
Control	-	329.69 ± 58.35
CTX	-	275.93 ± 28.19*
APS	160	379.26 ± 11.08* ∆∆
EAF-H	400	364.29 ± 14.73∆∆ ▲
EAF-M	200	328.45 ± 92.72
EAF-L	100	377.32 ± 12.52* ∆∆

Compared with control group,

* p < 0.05,

** p < 0.01;

compared with the model group,

∆ p < 0.05

∆∆ p < 0.01;

compared with APS group,

▲ p < 0.05

▲▲ p < 0.01.

In Table 6 the effect of serum hemolysin levels in immunocompromised mice can observed. Compared with the CTX group, the HC_50_ of all experimental groups were significantly increased (p < 0.01), and the three-dose EAF groups were notably different from APS group (p < 0.01), reaching similar levels to the control group.

**Table 6:** Effect of EAF on the level of hemolysin production in immunocompromised mice ( ± s, n = 6).

Groups	Dose (mg·kg^–1^)	Half hemolysis value (HC_50_)
Control	-	363.52 ± 6.35
CTX	-	248.09 ± 34.06**
APS	160	301.03 ± 8.00** ∆∆
EAF-H	400	344.53 ± 24.45∆∆ ▲▲
EAF-M	200	343.69 ± 32.62∆∆ ▲▲
EAF-L	100	365.71 ± 28.86∆∆ ▲▲

Note: Compared with control group,

* p < 0.05,

** p < 0.01;

compared with the model group,

∆ p < 0.05

∆∆ p < 0.01;

compared with APS group,

▲ p < 0.05

▲▲ p < 0.01.

## Discussion

For the first time, the present study investigated the immunomodulatory effects of *Blaps rynchopetera* extract. Macrophages are multifunctional immune cells that play a key role in all aspects of the body’s immune response^25^^,^ 26. Phagocytosis is one of the basic functions of macrophages. Therefore, an experimental model of immune activity of peritoneal macrophages as target cells was constructed to assay the immunomodulatory effects of the extract *in vitro* 25.

It has been previously shown that polysaccharides stimulate macrophage proliferation with low cytotoxicity^27^^,^ 28. Under the stimulation of LPS, the phagocytosis of macrophages is enhanced, suggesting that the peritoneal macrophages are activated and the innate immune function is enhanced. In this study, macrophages were stimulated with samples alone and in combination with LPS respectively to observe the effects on macrophages, and the phagocytic activity was evaluated by neutral red method. The results of the study indicated that all components can individually enhance the phagocytic ability of mouse peritoneal macrophages. After combined stimulation with LPS, the chloroform fraction and EAF showed that they could further increase the phagocytic ability of macrophages after LPS prestimulation to a certain extent.

*In vivo*, the effects of EAF on the immune function of normal and immunocompromised mice were further investigated in terms of both cellular and humoral immunity. Astragalus polysaccharide is an extract of *Astragalus membranaceus*, which is rich in nutrients and has pharmacological effects such as enhancing immunity and improving antioxidant capacity. It has been widely used in clinical medicine^29^. Studies have shown that APS can enhance the cellular and humoral immunity of animals by promoting the development of immune organs and enhancing the functions of T cells, B cells and natural killer cells^30^. In this study, APS was used as a positive control and CTX was used as a chemo-inducing agent in an immunosuppressive animal model. After CTX injection, spleen and thymus index decreased, peripheral blood immune cells showed a downward trend, and the secretion level of hemolysin decreased^31^. The spleen is the main organ of humoral immunity, and the thymus is the main organ of cellular immunity. The thymus and spleen indexes can reflect the overall immunity level of the body to a certain extent. The change of the number of peripheral blood cells can reflect the change of immune function^23^. Serum hemolysin is an important index to evaluate humoral immunity^32^.

The results of this study show that APS has a significant effect on both normal and immunocompromised mice. It is noteworthy that EAF could significantly increase the immune organ indexes, the level of peripheral blood immune cells, and the half hemolysis value for immunocompromised mice. Conversely, it has a much smaller influence on normal mice. These results indicate that EAF has a remarkably protective effect on immunocompromised mice induced by CTX, and the regulatory mechanism may be different from that of APS.

The test results of the normal group for both batches of experiments are also quite different, especially for the immune organ index. This reminds the necessity for using animals from the same batch in related subject research in future studies.

## Conclusions

In this study, experiments using neutral red assay revealed that all fractions can individually enhance the phagocytic ability of mouse peritoneal macrophages. The chloroform and EAF had a tendency to enhance the phagocytic ability of mouse peritoneal macrophages under combined stimulation with LPS. While EAF does not have a significant effect on the immune function of normal mice, it can significantly improve the state of low immunity caused by CTX, increase the level of immune cells, and enhance humoral immune function. Ethyl acetate fraction can regulate the balance of the abnormal immune state by stimulating immune organs and immune cells to produce immune response.
